# Acupuncture Effect and Central Autonomic Regulation

**DOI:** 10.1155/2013/267959

**Published:** 2013-05-26

**Authors:** Qian-Qian Li, Guang-Xia Shi, Qian Xu, Jing Wang, Cun-Zhi Liu, Lin-Peng Wang

**Affiliations:** Acupuncture and Moxibustion Department, Beijing Hospital of Traditional Chinese Medicine affiliated to Capital Medical University, 23 Meishuguanhou Street, Dongcheng District, Beijing 100010, China

## Abstract

Acupuncture is a therapeutic technique and part of traditional Chinese medicine (TCM). Acupuncture has clinical efficacy on various autonomic nerve-related disorders, such as cardiovascular diseases, epilepsy, anxiety and nervousness, circadian rhythm disorders, polycystic ovary syndrome (PCOS) and subfertility. An increasing number of studies have demonstrated that acupuncture can control autonomic nerve system (ANS) functions including blood pressure, pupil size, skin conductance, skin temperature, muscle sympathetic nerve activities, heart rate and/or pulse rate, and heart rate variability. Emerging evidence indicates that acupuncture treatment not only activates distinct brain regions in different kinds of diseases caused by imbalance between the sympathetic and parasympathetic activities, but also modulates adaptive neurotransmitter in related brain regions to alleviate autonomic response. This review focused on the central mechanism of acupuncture in modulating various autonomic responses, which might provide neurobiological foundations for acupuncture effects.

## 1. Introduction

 Acupuncture has been practiced for over 3000 years with beneficial clinical effects on many disorders [1]. There is sufficient evidence of the value of acupuncture to expand its application into conventional medicine and to encourage further studies of its physiological and clinical values [2]. According to traditional Chinese medicine (TCM), “acupuncture is believed to restore the balance between *Yin* and *Yang*.” This can be translated into the Western medicine terminology as “acupuncture modulates the imbalance between the parasympathetic and sympathetic activity [3].” Acupuncture has been effectively used in various autonomic nerve-related disorders, such as cardiovascular diseases, epilepsy, anxiety and nervousness, circadian rhythm disorders, polycystic ovary syndrome (PCOS), and subfertility [4–8]. It could influence some known indicators of autonomic activities, such as blood pressure [9–11], pupil size [12], skin conductance [13], skin temperature [14], muscle sympathetic nerve activities [15], heart rate and/or pulse rate [16], and heart rate variability [17, 18]. Acupuncture has been proposed to treat autonomic nerve-related diseases through modulating the imbalance between the sympathetic and parasympathetic activities [19]. Previous study has shown that changes in parasympathetic nervous activity are correlated with the amount of *De-Qi* (i.e., arrival of *Qi*) sensations during acupuncture manipulation [20]. On the other hand, the affecting degree of acupuncture on the autonomic nerve is still unknown because part of the acupuncture effects is dependent on the *De-Qi* sensation [21].

A literature review was conducted using PubMed, EBSCOhost, and the China National Knowledge Infrastructure (CNKI). Keywords used in the searching were “acupuncture,” “brain” or “cerebrum” and “sympathetic,” “vagus,” “autonomic,” or “parasympathetic.” Articles were collected from December 2007 to present in each database. The identified 44 publications in this search were related to acupuncture basic study and central autonomic regulation. Among these 44 articles which met the criteria, 35 articles are in English and 9 articles are in Chinese. In this review, the underlying central mechanism of acupuncture-induced autonomic modulation is discussed based on basic studies that have been published in the past 5 years. We will, in particular, focus on two aspects as follows: (1) the brain region which plays an important role in initiating autonomic responses during acupuncture; (2) neurohumoral autonomic modulation of acupuncture in the central autonomic nerve system (ANS). 

## 2. Acupuncture Effect and Central Autonomic Structures

**Figure 1:**
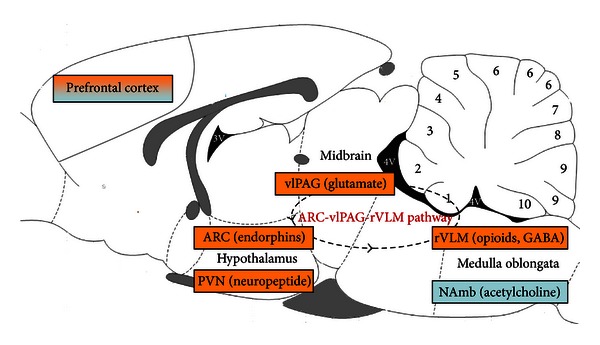
Acupuncture autonomic regulation mechanism. Blue indicates the area involved in acupuncture parasympathetic regulation. Orange indicates the area involved in acupuncture sympathetic regulation.

Several studies have demonstrated that the autonomic dimension of the acupuncture stimulation was mediated by a mesencephalic and brainstem network [22, 23] (Figure 1), which is comprised of the hypothalamus, medulla oblongata, ventrolateral periaqueductal gray, and the dorsomedial prefrontal cortex. All of these areas are involved in the autonomic regulation [24–26]. 

### 2.1. Hypothalamus

Hypothalamus is the most important brain center that controls the ANS [27]. As the site of autonomic regulation, hypothalamus has been proved to be involved in the pathway of electroacupuncture (EA) attenuating sympathetic activity. Impulses generated in sensory fibres in the skin connect with interneurons to modulate activities of the motoneurons hypothalamus to change autonomic functions [28]. Increased sympathetic activity in hypertension may act as a stimulus for nitric oxide (NO) release in the hypothalamus. EA application on ST36 could effectively modulate the activity and expression of neuronal nitric oxide synthase (nNOS) in the hypothalamus of spontaneously hypertensive rats (SHR). The effect may through its connections to sympathetic and parasympathetic nervous system and also through its control of endocrine organs [29]. However, which part of the hypothalamus that participates in the mechanism of action is still remained unclear. Effects on decreased neuropeptide Y (NPY) production due to stimulation on the paraventricular nucleus (PVN) of hypothalamus [30] is one of the several hypotheses which have been proposed in the literature regarding the action mechanism. The PVN of hypothalamus is a cell group that plays an important role in the regulation of sympathetic vasomotor tone and autonomic stress responses [31, 32]. Acupuncture could decrease NPY [33] and corticotropin-releasing hormone [34] expressions in the PVN and produce some specific effects on suppressing the sympathetic outflow in response to chronic stressors [35].

Arcuate (ARC) nucleus projects to other brain regions that regulate the sympathetic outflow include the dorsomedial hypothalamus, midbrain periaqueductal grey, rostral ventrolateral medulla (rVLM), and the nucleus of the solitary tract [36]. Neurons in the ARC nucleus projecting to the rVLM potentially participate in EA inhibition of reflex cardiovascularsympathoexcitation [37]. Ventrolateral periaqueductal gray (vlPAG) projections from the ARC are required for EA regulation of sympathoexcitatory presympathetic rVLM activity and the cardiovascular excitatory reflex responses, while a direct pathway between the ARC and rVLM might serve as a source of endorphins for EA cardiovascular modulation. 

### 2.2. Medulla Oblongata

Specific regions of the medulla oblongata mediate central control of autonomic function. In the central nervous system (CNS), the rVLM is an important part of the sympathetic efferent limb of cardiovascular reflex activity and, as such, it is important in the maintenance of arterial blood pressure [38]. It projects to the intermediolateral columns of the thoracic spinal cord, which is the origin of sympathetic preganglionic neurons [39]. Inhibition of neuronal function in this nucleus results in large decreasing of blood pressure [40]. EA could inhibit cardiovascular autonomic responses through modulating rVLM neurons [41, 42]. Moreover, opioids and gamma-aminobutyric acid (GABA) participate in the long-term EA-related inhibition of sympathoexcitatory cardiovascular responses in the rVLM [43]. Activation of the nucleus raphe pallidus (NRP) attenuates sympathoexcitatory cardiovascular reflexes through a mechanism involving serotonergic neurons and 5-HT1A receptors in the rVLM during EA. Serotonergic projections from the NRP to the rVLM contribute to the EA-cardiovascular responses [44]. 

The nucleus ambiguus (NAmb), located in the ventrolateral division of the hindbrain, is considered to be an important site of origin of preganglionic parasympathetic vagal motor neurons that ultimately regulate autonomic function through the releasing of acetylcholine [45]. The recent study of that neurons colabeled with c-Fos and choline acetyltransferase (ChAT) were activated in the EA-treated animals instead of sham EA group indicates that some NAmb neurons activated by EA are preganglionic vagal neurons [18]. It is suggested that stimulation on a special acupoint is crucial to achieve modulate effect on autonomic function by activating NAmb neurons. It is consistent with TCM theory that genuine acupoints treatment is more effective than nonacupoints treatment based on specific physiological effects related to meridians and collections of meridian *Qi*. 

### 2.3. Midbrain

 Ventrolateral periaqueductal gray (vlPAG) is an essential midbrain nuclei that process information from somatic afferents during EA [46]. Caudal vlPAG is a significant region in the long-loop arcuate-rVLM pathway for the EA-cardiovascular response, while the rostral vlPAG plays a major role in the reciprocal arcuate-vlPAG pathway that helps to prolong EA-cardiovascular modulation [47]. Excitation of vlPAG neurons enhances the arcuate response to splanchnic stimulation, while blockade of vlPAG neurons limits excitation of arcuate neurons by EA. These observations indicate that EA-induced excitation of arcuate neurons requires input from the vlPAG, and the reciprocal reinforcement between the midbrain and the ventral hypothalamus serves to prolong the influence of EA on the baseline blood pressure [48]. 

### 2.4. Dorsomedial Prefrontal Cortex (DMPFC)

The prefrontal cortex (PFC) is vital for mediating behavioral and somatic responses to stress in the autonomic centers via projections [49]. A near-infrared spectroscopy (NIRS) study found that the right PFC activity predominantly modulated sympathetic effects during a mental stress task [50]. Acupuncture stimulation might decrease sympathetic activity and increase parasympathetic activity through its inhibitory effects on dorsomedial PFC activity [51]. This might be beneficial to treat chronic pain induced by hyperactivity of the sympathetic nervous system. However, Sakatani et al. found no significant correlation between the PFC activity and ANS function during acupuncture. One of the possible explanations of the poor correlations might be that the PFC activity induced by acupuncture is not closely linked with ANS function [52].

## 3. Acupuncture Effect and Neurohumoral Modulation

Some neurotransmitters, including serotonin, opioid peptides, catecholamines, and amino acids in the brain appear to be participated in the modulation mechanism of acupuncture for certain ANS [53, 54]. 

### 3.1. Endogenous Opioids

EA was able to restore the impaired gastric motility and dysrhythmic slow waves by enhancing vagal activity, which was mediated via the opioid pathway [55, 56]. Ameliorating effects of EA at ST-36 on gastric motility might activate the central opioids that, in turn, inhibit sympathetic outflow [57]. Although acupuncture produced significant heart rate decreases in pentobarbital-anesthetized rats, this response is related to the activation of GABAergic neurons instead of opioid [58]. This opinion is proved by another study, which indicates that an opioid receptor-mediated transmission is not responsible for the present bradycardiac response induced by acupuncture-like stimulation [59]. These views suggest that acupuncture treatment on different diseases may be mediated by different neurotransmitters, which is in accordance with holistic view of acupuncture treatment in TCM theory. 

EA activates enkephalinergic neurons in several brain areas that regulate sympathetic outflow, including the arcuate nucleus, rostral ventrolateral medulla, raphé nuclei, among others [60, 61]. Consistent with this, Li et al. [62] found that EA at P5-P6 transiently stimulates the production of enkephalin in a region of the brain, which regulates sympathetic outflow. It is suggested that a single brief acupuncture treatment can increase the expression of this modulatory neuropeptide. The *β*-endorphin is a key mediator of changes in autonomic functions [63]. Acupuncture may hypothetically affect the hypothalamic-pituitary-adrenal (HPA) axis by decreasing cortisol concentrations and the hypothalamic-pituitary-gonadal (HPG) axis by modulating central *β*-endorphin production and secretion [64]. Some reports have also shown that a negative perception of acupuncture might produce enhanced sympathetic activation to the acupuncture stimulus [65], which may be mediated through endorphin pathway [66]. It is conceivable that a specific neuroendocrine-immune network is crucial to the produce of acupuncture therapeutic effect. Further studies are required to reveal involved molecules and underlying mechanisms.

### 3.2. Amino Acids

Amino acid sensors could regulate the activity of vagal afferent fibers [67]. Amino acids are directly involved in signaling the vagus pathway in the ARC [68]. Recent studies have shown that vesicular glutamate transporter 3 (VGLUT3) in the ARC neurons [69, 70] and vlPAG [60, 71] were activated by EA at the P5-P6 acupoints. Glutamate only partially but significantly contributes to the activation of ARC-vlPAG reciprocal pathways during EA stimulation of somatic afferents [47]. In addition, reduction of GABA release disinhibits vlPAG cells, which, in turn, modulates the activity of rVLM neurons to attenuate the sympathoexcitatory reflex responses [46]. EA modulates the sympathoexcitatory reflex responses by decreasing the release of GABA in the vlPAG [43], most likely through a presynaptic CB1 receptor mechanism [72]. Studies conducted so far on amino acids suggest that glutamate and GABA are involved in the mechanism of acupuncture for autonomic alteration. This response is closely related to vlPAG.

### 3.3. Nerve Growth Factor (NGF)

The NGF is a neurotrophin, which regulates the function and survival of peripheral sensory, sympathetic, and forebrain cholinergic neurons. It could modulate sensory and autonomic activity as a mediator of acupuncture effects in the CNS [73]. The therapeutic potential of EA could modulate the activity of the ANS by a long-lasting depression of the sympathetic branch, which is associated with a peripheral downregulation of NGF in organs. Mannerås et al. [74] found that EA could effectively improve PCOS-related metabolic disorders, alter sympathetic markers [75], and normalize the DHT-induced increase of mRNA^NGF^. The data on EA/NGF interaction in PCOS models further suggested that the decrease of NGF expression in peripheral organs could benefit EA to modulate the activity of the ANS [76]. Although NGF in organs has been proved to be associated with the acupuncture effect on ANS, there is a lack of sufficient evidence to demonstrate the relationship between acupuncture effect and NGF in central autonomic nerve system. 

## 4. Conclusion

Emerging evidence indicates that acupuncture treatment not only activates distinct brain regions in different kinds of diseases caused by imbalance between the sympathetic and parasympathetic activities, but also modulates adaptive neurotransmitter in related brain regions to alleviate autonomic response. However, it is not clear whether different pathway is activated by specific acupoint, such as local points and distant points, or the autonomic regulation effect of acupoints from different meridians. Further rigorous RCTs are required for the study of this topic. It enables us to understand the importance of acupuncture therapy in the autonomic regulation. Then, acupuncture can be used in the treatment of various autonomic disorders as a novel alternative therapy.
